# By the Way

**Published:** 1910-12-24

**Authors:** 


### By the Way.

### THE HEIGHT OF ANTISEPSIS.

At a recent duel the adversaries had already crossed swords and were waiting for the signal to begin, when a voice was heard, "One moment! Gentlemen!" A pause, while the onlookers hoped for a reconciliation. But alas! it was only the surgeon in attendance. Imbued with modern ideas, our *confrère* drew from his pocket a carbolic solution and carefully immersed in it the points of the rapiers. Then, in the tone of one who has done his duty, "Go on now, gentlemen," said he, "you may kill one another, but you are at least free from the possibility of a purulent infection!" —*Le Petit Moniteur de la Médecine*.

### THE ROYAL TOUCH.

A special article appeared in The Hospital for August 13, 1910, on "The Mystery of the Royal Touch," and the following anecdote from the Memoirs of René-Louis de Voyer, Marquis d'Argenson, has some bearing on the same subject. The monarch referred to is Louis XV., who had just completed his minority. "1723. At the coronation of the King, at Rheims, a man from Avesnes, suffering from a horrible form of scrofula, went up to be touched by his Majesty. He was absolutely cured. Having heard of this, I caused inquiries to be made into his previous and subsequent health. This done, I forwarded proofs of the miracle to M. de la Vrillière, Secretary of State for the Province. I thought to obtain great praise for my zeal in the matter of the Royal prerogative. I received, however, a dry letter in reply, informing me that nobody doubted that our kings possessed this power. But I was nevertheless certain that everything had been read to the King, who, child as he was, yet liked to hear it said that he had wrought this miracle."

### A PRECURSOR OF THE ABOLITIONIST.

In view of the present activity of societies the aim of which is the suppression of the drink traffic, the following notice from the columns of *L'Intermédiaire des Chercheurs et des Curieux* may be of interest. It will be seen that abolitionists existed even in the seventeenth century, a time when heavy drinking was common and looked upon as quite natural. Moreover, they practised what they preached, as the following will show. The notice consists of a printed leaflet at the top of which appears the arms of France, a signed autograph dated February 3, 1683, bringing it to a close. "We forbid with great emphasis all sorts of persons, whatever be their quality and condition, as also the magistrates of towns in which a garrison may be quartered, to establish any canteen for the sale of brandy, and to sell that commodity to any horseman, dragoon, and soldier, at any price and under any pretext whatsoever, the penalty being a fine of 150 livres as well as all expenses, damages, and interests, half of which shall go to the informer, and the other half to the Capucin Convents nearest situated to each town, citadel, or castle in which the present order shall have been contravened. Which order shall take force, be published, and affixed in such places as there is need of it, to the end that nobody shall plead ignorance as a cause of its infraction."

### THOSE GOOD OLD TIMES.

The following appears in the collection "Guy Patin," which has been recently published by Dr. Pierre Pic: "March 25, 1657. M. the First President is here, very ill: he has been bled for the sixth and seventh time; at first he was unwilling to be bled, but at present he is only too anxious to have it done, and says that he is fully aware that he can be cured only in that way. *Tandem bona causa triumphat.*" . . . "M. the First President was again bled yesterday for the tenth time(!) The doctors who surround and beset him deliberate about giving him some slight purgative, feeling that they have arrived at the eighth day. Finally they give him one said to be composed of cassia, senna, and manna. *A quo longe deterius habuit* with the result that it was necessary to bleed him again this evening *ne suffocaret.*" Eleven bleedings in eight days! This First President had a rich nature!—*La Gazette Médicale Belge*.

### THE THIRST OF NATIONS.

The *Strassburger Post* has recently devoted space to a comparative statistical study of thirst as shown by the amount of liquor consumed by the inhabitants of various European States. The task is complicated by the varieties of liquid refreshment which are met with in different countries and which are common to few. It appears, however, that the Dane drinks on an average 104 litres of beer, very little wine, but 24 litres of brandy each year. The Swede is satisfied with 56 litres of beer and nine of alcohol. The Norwegian is one of the most temperate of Northern nations, consuming but 31 litres of beer and three of brandy per head of population. The Russian takes 5 litres each of beer and vodka, whereas his ally the Frenchman needs 32 litres of beer, ten of brandy, and 108 of wine. John Bull imbibes 6 litres of whisky or gin, two of claret, and 152 of beer or stout. The Dutchman rests content with 38 litres of beer and eight and a half of brandy. His neighbour the Belgian is more capacious, being satisfied only with 221 litres of beer and nine of alcohol. The Austrian and the Hungarian each absorb 11½ litres of schnapps and sixteen of wine, the former needing in addition 80 litres of beer, while eleven will suffice for the latter. Of all the inhabitants of Europe the Italian is the one who drinks least beer, a mere 2 litres, and the least alcohol, 1¼ litre; he imbibes 98 litres of wine, however. As regards Germany, it would be almost unjust to talk of a mean. If one takes into account the whole extent of the Zollverein, including Luxemburg, the consumption per head amounts to 125 litres of beer, seven of wine, and six and a half of alcohol; but if each State is considered separately, one finds that the Northern German and the Alsatian are satisfied with 98 litres of beer, whereas 158 are necessary to the inhabitant of Baden, 169 to the Würtemburgian, and 240 to the Bavarian. Lastly, in comparing one great beer town with another, one finds that Berlin drinks 200 litres per head of inhabitants, Nuremberg 325, Frankfort 432, and Munich 570.—*Journal des Débats.*

### A RETROSPECT.

*Apropos* of the cases of plague reported in Suffolk, it is interesting to recall that a hundred and fifty years ago a false report was circulated that the plague had broken out in St. Thomas's Hospital, as will be observed by the following advertisement which appeared in the *London Gazette* at the time:—

"St. Thomas's Hospital, July 30th, 1760.—Whereas the town has been alarmed by a false and wicked report, that the plague is broke out in St. Thomas's Hospital; we the underwritten (in pursuance of an order of the grand committee of Governors held this day), do hereby certify, that the said report is absolutely without foundation; and that there are no other diseases among the patients, than what are usual in this and all other hospitals."

The statement was signed by the physicians, surgeons, and apothecary to the hospital.

According to an account in Ward's "Mems, Maxims, and Memoirs," the wicked report mentioned above spread a general consternation, and the demand for rue and wormwood in Covent Garden Market advanced the price of those articles almost 40 per cent., the gardeners' servants being employed night and day in taking those commodities to market. It is related by the same authority that about this time Messrs. Chandler and Smith, apothecaries, in Cheapside, had taken in a partner, and while the report of the plague prevailed these gentlemen availed themselves of the popular opinion and put a written notice in their windows of "*Four Thieves* vinegar sold here." Mr. Ball, an old apothecary, passing by and observing this, went into the shop. "What," said he, "have you taken in another partner?" "No." "Oh! I beg your pardon," replied Ball, "I thought you had, *by the ticket* in your window."

### RAISINS AND HYGIENE.

The manipulations to which raisins are submitted in the Orient before shipment are of the simplest. After having been passed through a solution of potash, the grapes are merely dried in the sun. For the last few years it has been customary, owing to the public having become more fastidious, to remove the fruit from the stalks before distribution. This innovation greatly facilitates the task of the confectioners, but it presents certain inconveniences from the point of view of hygiene. The process is carried out in large rooms, where Greek and Turkish women, for the most part aged, work. The greatest liberty is allowed them in the choice of methods they employ, and there are different schools of thought. Whereas some make use of their nails, most employ their teeth, or rather what remains of them, either gripping the stalk in their mouths and keeping the fruit in their hands, or, on the contrary, seizing hold of the stalk in their hand and momentarily enclosing the fruit in the buccal cavity, to expel it immediately into their apron. It would seem that this last procedure is the one which gives the maximum of speed and consequently of wages, for the women are paid by piece-work. But unfortunately it is not without its drawbacks, for the potash with which the fruit is impregnated is apt to set up troublesome stomatitis and gingivitis. The doctor states that after learning of this practice his taste for plum-cake has received a set-back. He has been filled with pity for the buccal mucous membrane of these poor women, and has commanded his cook to pay great attention to the toilet of such Corinth raisins as she may find it necessary to make use of, and, moreover, to restrict their appearance to the absolute minimum compatible with culinary exigencies.—*Revue de Thérapeutique Médico-Chirurgicale.*

### ANCIENT ROMAN TOOTHACHE CURES.

If it be true that ancient remedies are always the best, it may, says the *Journal des Débats*, be of interest to those afflicted with dental troubles to know how the ancient Romans dealt with such ills. The Quirites recognised two types of treatment, the magical and the medical. The following are some of the prescriptions advised by the magicians. Take the head of a dog that has died of rabies, mix the ash with oil of Cyprus, and inject the product into the ear of the affected side. A water-snake's vertebra will serve to scarify the gum provided that it be obtained from a white-skinned snake. Or for the same purpose may be used a lizard's frontal bone obtained when the moon is full, or, if that fail, a chicken bone will do, provided that it be dried in a hole in a wall and thrown away immediately after use. It is good treatment to inject into the ear oil of lemon in which has been macerated either mallow bugs or sparrows' dung, even should this last give rise to itching. A worm fed on a particular herb, or a cabbage caterpillar can conveniently be placed in a hollow tooth, but it is equally simple to chew an adder's heart. Prevention being better than cure, a sovereign preventative will be found in the eating of two rats per month. Pliny's prescriptions, on the other hand, are frankly more medical. In the vegetable kingdom he recommends the roots of the plantain, verbena, birth-wort and hyssop, any of which may be chewed or taken as a decoction or infused in wine or vinegar after a preliminary washing in sea-water. The infusion should be held in the mouth while tepid, or some of it may be poured down the nostril of the sound side. Glue, ox-gall, and goat's milk form a perfect elixir, and the mouth should afterwards be rinsed out with the juice of stewed pomegranates. Burnt horn, the ash of the heads of a wolf or of a hare or of a mouse, or that of a pig's foot, all make useful tooth-powders. Lastly, to relieve pain the animal kingdom furnishes him with the *vivar*, the bones of which fish are used to scarify the gum, and more especially with the frog, of which the uses are many. It may be cooked in vinegar and employed as a gargle, or the animal may be suspended from the jaw to draw out the pain. Pliny forgets, unfortunately, to mention how long it should be left suspended.

### A FORGOTTEN WORTHY.

Jean Rene Sigault performed the first operation of symphysiotomy on the evening of October 1, 1777, assisted by Dr. Leroy, President of the Paris Faculty of Medicine. The patient wife of a soldier named Souchot had been confined on five occasions previously of a dead child. The operation was a complete success, but the glory of the inventor was short-lived, as a few subsequent failures gave opponents the opportunity they were seeking of decrying the method, so that in a short time it came to be regarded almost as a criminal operation and was banished from among recognised methods of treatment. Alone in Europe was it upheld by the Italian school of Naples. We are indebted to the researches of Drs. Monprofit and Labesse for details concerning the career of this once-illustrious but now almost forgotten French obstetrician. In 1768 Sigault had presented a thesis on symphysiotomy to the Academy of Surgery, but this was rejected. The University of Medicine of Angers proved, however, more hospitable, and in 1773 he received his degree of Doctor of Medicine for a work entitled *An in partu contra naturam sectio symphyseos ossium pubis sectione caesaria promptior et tutior.* He then proceeded to Paris, where he submitted another work on a different subject to the Faculty of Medicine, as is shown by the following inscription set off against his new thesis: *Johannes Renatus Sigault, Divionoeus, Doctor-Medicus Andegavensis, nec non saluberrimae Facultatis Medicinae Parisiensis baccalaureus, A.R.S. 1776.*

It was in the following year that his pioneer operation was performed. Its success led to unbounded enthusiasm on the part of the Academy of Medicine and the Faculty, which at a memorable meeting unanimously passed the following resolutions: (1) "That the report of the operation be printed at once in the name and at the expense of the Faculty, and then distributed, not only to all doctors, native and foreign, but also to the gracious monarch now reigning, to the princes and magistrates, in order that all the world might be made aware of the discovery of this new method of saving mothers and children. (2) The Faculty regrets being unable to give a pension to the woman Souchot, but grants her a relief of 360 livres. It promises her its services and good offices, and also to carry its respectful prayers on her behalf to the feet of his Gracious Majesty, and to solicit from the Ministers and all ranks of citizens a pension for this brave woman who submitted herself to a new operation. (3) The Faculty is unable to recompense in a worthy manner the inventor of a discovery so useful to humanity, but, as a pledge of its gratitude and admiration, it orders that the silver token of the Dean be engraved with the following inscription: *Sectio: symphys: oss: pub: 1768 Invenit, proposuit; 1777 Fecit feliciter J.-R. Sigault, D.M.P., Juvit Alph. Le Roi D.M.P.*" It is remarkable, in the presence of these acclamations of his contemporaries, who, ignorant of antiseptics, were only able to appreciate with difficulty the true merit of Sigault's discovery, that his name should have quickly fallen into oblivion; but such is fame!

### THE GREGORIAN CALENDAR.

When, in the sixteenth century, the influence of emancipation and a salutary return towards a beautiful and healthy antiquity delivered arts and letters from the deep slumber in which the Middle Ages had wrapped them, medicine, for eight centuries subservient to the barbarous doctrines of the Arabs, also took wings and wished to pierce the thick veil in which prejudice and superstition had enwrapped it. Alchemy and astrology were, in 1500, inseparable from the art of healing, and the greatest physicians had yielded more or less to the troublesome influence of this doctrine. It was even a prevalent custom to consult calendars containing the announcement of the time and the interpretation of the stars from the point of view of their influence on disease, calendars compiled by doctors, and which largely helped to propagate a belief in astrology. The Renaissance came to upset all these absurd practices and to hasten the revival of Hippocratic doctrines long since abandoned.

Not everything was lost to science, however, in this long epoch of ignorance and arrested civilisation, for the doctors acquired, after several centuries of astrological observations, some real knowledge, not of the influence of the stars on disease, but of astronomy and cosmography. It was thus that one of them, at the end of the sixteenth century, brought to notice the so-called Gregorian calendar. The name of this learned physician deserves to be handed down to posterity; how many people have even heard it? Voltaire tells us what it was. "It was not a question in the times of Gregory XIII. of attempting to explain the cause of this precession of equinoxes, but of putting some kind of order to the confusion which began definitely to trouble the civil year. Gregory caused all the celebrated astronomers of Europe to be consulted. A physician named Lilio, born at Rouen, had the honour of supplying the simplest and easiest method of re-establishing the order of the year such as is found in the new calendar; all that was necessary was to take away ten days from the year 1582, in which they then were, and to prevent similar derangement in future centuries by simple precautions. This Lilio has since then been lost sight of, and the calendar bears the name of Gregory, in the same way that the name of Sosigenes was obscured by that of Cæsar. Things were not done in this way among the Greeks; the glory of an invention remained with the artist." Our astronomical *confrère* merited this rehabilitation, and it is but right that present and future generations should know his name—Dr. Ravon, de Chazelle, in *La Médecine Littéraire.*

